# Unraveling Urban Form and Collision Risk: The Spatial Distribution of Traffic Accidents in Zanjan, Iran

**DOI:** 10.3390/ijerph18094498

**Published:** 2021-04-23

**Authors:** Mohsen Kalantari, Saeed Zanganeh Shahraki, Bamshad Yaghmaei, Somaye Ghezelbash, Gianluca Ladaga, Luca Salvati

**Affiliations:** 1 Department of Human Geograhy and Spatial Planning, Faculty of Earth Sciences, Shahid Beheshti University, 1613778314 Tehran, Iran; mo_kalantari@sbu.ac.ir; 2 Department of Human Geography, University of Tehran, 1613778314 Tehran, Iran; 3 Department of Remote Sensing and Geographical Information Systems, Faculty of Earth Sciences, Shahid Beheshti University, 1613778314 Tehran, Iran; b_yaghmaei@sbu.ac.ir; 4 Faculty of Earth Sciences, Shahid Beheshti University, 1613778314 Tehran, Iran; s_ghezelbash@sbu.ac.ir; 5 Istituto Nazionale per l’Assicurazione Contro gli Infortuni sul Lavoro (INAIL), Viale Vincenzo Verrastro 3/C, I-85100 Potenza, Italy; g.ladaga@inail.it; 6 Department of Economics and Law, University of Macerata, Via Armaroli 43, I-62100 Macerata, Italy; luca.salvati@unimc.it

**Keywords:** urban transportation, traffic accidents, spatiotemporal interactions, hazardous locations

## Abstract

Official statistics demonstrate the role of traffic accidents in the increasing number of fatalities, especially in emerging countries. In recent decades, the rate of deaths and injuries caused by traffic accidents in Iran, a rapidly growing economy in the Middle East, has risen significantly with respect to that of neighboring countries. The present study illustrates an exploratory spatial analysis’ framework aimed at identifying and ranking hazardous locations for traffic accidents in Zanjan, one of the most populous and dense cities in Iran. This framework quantifies the spatiotemporal association among collisions, by comparing the results of different approaches (including Kernel Density Estimation (KDE), Natural Breaks Classification (NBC), and Knox test). Based on descriptive statistics, five distance classes (2–26, 27–57, 58–105, 106–192, and 193–364 meters) were tested when predicting location of the nearest collision within the same temporal unit. The empirical results of our work demonstrate that the largest roads and intersections in Zanjan had a significantly higher frequency of traffic accidents than the other locations. A comparative analysis of distance bandwidths indicates that the first (2–26 m) class concentrated the most intense level of spatiotemporal association among traffic accidents. Prevention (or reduction) of traffic accidents may benefit from automatic identification and classification of the most risky locations in urban areas. Thanks to the larger availability of open-access datasets reporting the location and characteristics of car accidents in both advanced countries and emerging economies, our study demonstrates the potential of an integrated analysis of the level of spatiotemporal association in traffic collisions over metropolitan regions.

## 1. Introduction

Road accidents are demonstrated to be one of the major sources of injuries and fatalities worldwide [1,2,3,4]. Every year, more than 1.24 million people die on the road, and 50 million people suffer non-fatal injuries [5]. Traffic injuries are continuously increasing, representing the ninth cause of death in 1999, and being expected to rank third by 2020 [6,7]. Traffic crashes have been reported to be a leading injury-related causes of death among people aged between 15 and 29 years [8,9,10]. Moreover, these events account for a loss of nearly 1% of Gross National Product in many countries [11]. The share of fatal traffic accidents in total accidents is particularly high in low- and middle-income countries [12].

Identification of hazardous locations is one of the most important issues when preventing (or containing) traffic accidents. Transportation planners usually identify ‘risky’ places in an attempt to enhance road safety by undertaking risk reduction actions [13]. The recognition of ‘risky’ locations or ‘safety black zones’ is the initial step of a comprehensive ‘traffic safety’ analysis [14]. This investigation stage identifies target locations as the earliest action of a more comprehensive road safety management strategy [15]. Background information provides a scientific basis for traffic management and regulation, optimizing ‘safety black zones’. To improve safety, it is necessary to consider every component of the urban traffic system. Detecting ‘safety black zones’ and analyzing the spatiotemporal factors affecting car accidents at these sites—as well as undertaking a series of measures to regulate vehicle traffic at these locations—may reduce the risk of collisions, saving human and financial resources [16].

Since local mobility and safe transportation are key elements in the development of safer traffic environments [17], analysis of spatiotemporal interactions among specific events has become relevant in urban planning and management [18,19,20,21,22,23]. Spatial and temporal correlations and relations of traffic crashes have been observed across different time scales, i.e., years, months, weeks, days, and even hours [15,21,24,25]. Spatiotemporal correlation profiles in car collisions can be explained with the assumption that several traffic-related factors exhibit some spatiotemporal features [26,27,28], e.g., since neighboring roads have similar traffic flow characteristics and, consequently, accidents are spatially clustered over defined temporal units. Results of a spatiotemporal analysis of traffic accidents may become a useful guide for accident management and prevention [20,29,30,31,32].

Empirical models have more frequently incorporated a detailed analysis of spatiotemporal interactions among traffic collisions with the final aim at improving precision of statistical estimates and higher prediction capability [33,34,35,36]. This goal can be realized with the implementation of a spatiotemporal investigation of the intrinsic risk associated with different travel modalities [18]. Assuming spatiotemporal interactions as a basic factor shaping collision density [37,38,39,40], different approaches have been proposed for explicit analysis of processes interacting over space [13,14,15,16,17,18,19,20]. Given the benefits associated with a comprehensive knowledge of temporal and spatial correlation among individual events [31,32,33], crash (frequency) data were usually aggregated over space and time [16], which may produce unobserved heterogeneity, as crashes that occur close in space or time are likely to share some unobserved characteristics [21,41,42,43].

Methodologies evaluating areal data (e.g., density of collisions over local administrative domains, such as counties, districts, municipalities and boundaries of local communities/settlements) are relatively common [44], implementing both spatially implicit and spatially explicit methodologies [45]. Use of spatially explicit approaches in accident analysis and prevention intensified in the last two decades [46], with the advent of open-access data, statistical software, and customary geographical information systems [47]. For instance, descriptive statistics, mapping, and spatial autocorrelation coefficients were extensively adopted when exploring the spatial distribution of road collisions [48,49,50]. Refined, geographically weighted estimation methodologies were also occasionally presented and adopted for analysis of specific cases [51]. Accident seasonality (over days, months, and years) was also evaluated [52], confirming the importance of both analysis’ dimensions (time and space). 

More recently, Aguero-Valverde and Jovanis [6] developed a spatiotemporal model for analysis of fatal accidents and injury crashes at the county level under a Bayesian framework, proposing a flexible approach that evaluates the hierarchical nature of collision data [37]. Waizman et al. [53] introduced a dynamic micro-simulation approach particularly useful in prediction and prevention of road collisions at specific hotspots. Despite a long tradition of study, multivariate spatiotemporal models accounting for unobserved heterogeneity [54] separately in spatial, temporal, and joint spatiotemporal correlations among different severity levels, were more occasionally developed [55,56,57,58]. Empirical results of earlier studies confirmed the intrinsic superiority of spatiotemporal models [59] over alternative random effects and more simplified exploratory approaches [18,60,61]. Given the issues mentioned above, road networks safety and the important (and sometimes irreversible) physical, psychological, social, and financial damages of collisions, justify further investigation of spatiotemporal approaches informing (evidence-based) strategies for the reduction of traffic accidents in urban areas [62].

Iran has one of the highest crash-related death rates in emerging economies (34 cases out of 100,000 inhabitants) [63]. One out of four casualties in Iran is caused by traffic accidents [8]. Human failure was reported as the primary cause of death in more than 70% of such events [64], and the cost of road traffic injuries accounts for 2.2% of gross domestic product [26]. According to national Forensic Medicine Statistics, one person dies in a traffic accident every 24 min in Iran and over 200 thousand deaths and injuries happen annually within the road network (extending only 117 thousand kilometers and including 1017 urban centers). Official statistics indicate that the number of car accidents in the country has decreased slightly in recent years. While the slow decline in road collisions seems to be a promising argument, Iran is still one of the most vulnerable countries in the world as far as traffic accidents are concerned [26]. Collision density is increasingly associated with urbanization, increasing population in residential settlements, economic growth, technological development, changing lifestyles, and concentration of private vehicles in metropolitan regions.

Being reflective of generalized dynamics typical of several cities in the Middle East, Zanjan—a medium-size city in Iran—has expanded road infrastructures because of the increase in car ownership and traffic load, as a result of massive (and mostly unplanned) urban development. Traffic accidents have been (and still are) a key transportation issue in Zanjan. According to the General Directorate of Roads and Urban Development of the Zanjan province, more than 350 people die each year in traffic accidents on provincial roads and more than 950 people are injured and taken to hospitals [65]. The high volume of private vehicles, the availability of nonstandard accesses to motorways, and the improper design of roads justify a spatially explicit analysis of urban accidents and the resulting injuries in this city. Based on these premises, the present study assesses spatiotemporal interactions among traffic accidents in the city of Zanjan, comparing results from Nearest Neighbor Indexes (NNI), Kernel Density Estimation (KDE), Natural Breaks Classification (NBC), and Knox’s model applied to individual records of collisions. A comparative analysis of such methodologies allows a spatially explicit investigation of the spatiotemporal association among traffic collisions, as a contribution to a refined strategy containing road accidents in densely populated cities.

## 2. Methodology

### 2.1. Study Area

**Figure 1:**
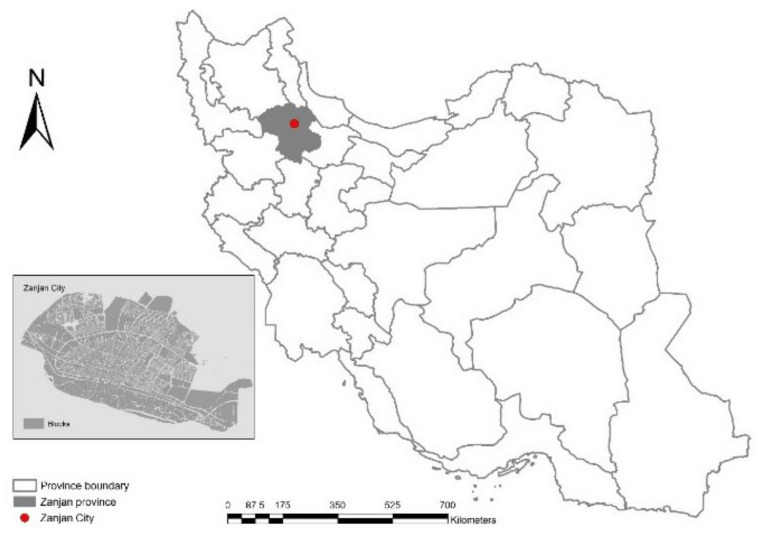
Location of Zanjan province in Iran (right) and a map of Zanjan city structure (insert).

The area investigated in our study corresponds to the administrative boundaries of Zanjan city (Figure 1), the capital of the Zanjan province, located along the Tehran-Tabriz road, at an average elevation of 1663 m above the sea level and with a resident population of more than 430,000 inhabitants (Source: Iranian Statistical Centre). Zanjan was developed on a narrow plain with a gentle slope from northeast to southwest, so that the elevation difference along the north–south gradient is about 100 m. Settlement growth in recent times has been concentrated toward the northern direction due to the topographical position of the area. 

According to the available demographic statistics, the total population of Zanjan amounted to 20,000 inhabitants in 1869, and increased to 39,450 inhabitants in 1941. According to the first official census, the city had 47,159 inhabitants in 1956. The last census (2016) enumerated a population of nearly 430,000 inhabitants (Table 1). Zanjan city faces with traffic problems due to the increased number of private cars. One of the main sources of traffic collisions is the ineffective system of public transportation. Only four bus lines and one minibus line circulate within the city boundaries. Private transportation was used in about 67 per cent of urban trips. 

### 2.2. Data Sources and Variables

A complete data set reporting road accidents (vehicle collisions) was adopted in the present study as obtained from the Zanjan Provincial Directorate of Traffic. This file includes all traffic accidents (*n* = 1943) occurred within the boundaries of Zanjan city from October 2014 to October 2015. Road accidents were geo-referenced and individual data were supplemented with basic information such as road, date, day of the week, and time. The basic cause of collision was finally recorded.

### 2.3. Statistical Analysis

A statistical strategy integrating different spatially explicit approaches was presented in this study with the aim of assessing the (intrinsic) relationship among road accidents in Zanjan city, considering together space and time dimensions. This strategy included (i) an automatic (non-parametric) estimation of collision density using Kernel algorithms, (ii) a nearest neighbor analysis of the spatial structure (clustering vs. randomness) of car accidents, (iii) a natural breaks algorithm identifying optimal classes describing the spatial distribution of car accidents in Zanjan, and finally (iv) a Knox test providing an inferential analysis of spatiotemporal associations among collisions in the study area. These approaches are described and discussed below in more detail. ArcGIS, SPSS, Excel and ClusterSeer2.5 software packages were used to analyze individual accident records.

#### 2.3.1. Kernel Density Estimation

Kernel Density Estimation (KDE) is a popular hotspot mapping methodology, converting point observations to a continuous density surface that summarizes the point distribution [65]. KDE was demonstrated to significantly outperform other statistical techniques in predictive hotspot mapping [17], being mainly used for determination of spatial point patterns such as geo-referenced location of road collisions. Assessing road accident hotspots using KDE has become increasingly popular because of its technical advantages over other hotspot detection approaches [19,30,43]. Unlike general clustering algorithms, grouping collision data in an unsupervised manner and determining a continuous density surface of road collisions are the main advantage of KDE [14]. The following KDE parameters were adopted in this study:

$${\hat{f}}_{n}=\frac{1}{nh\;}\overset{n}{\underset{i=1\;}{\sum }}k\;(\frac{x-R_{i}}{h}),\;\forall_{x}\in R$$

where *n* is the sample size, *h* is the bandwidth, *i* is the observation, and *R* is the set of real numbers, respectively. These values are required for application of Kernel functions [37] under the following conditions: 

Non-negativity: $K\left(x\right)>0,\forall_{x}\in R$

Symmetry: $K\left(x\right)=K\left(-x\right),\forall_{x}\in R$

Normalization:$\int_{+a??}^{-a??}K\left(x\right)=1$

#### 2.3.2. Nearest Neighbor Approach

Nearest-Neighbor Interchange (NNI) is a methodology testing for observations’ randomness or clustering in a given geographical area [66]. The NNI evaluates small-scale spatial interactions between individual collisions by analyzing, case by case, the nearest accidents in the data. With this test, the apparent distribution of empirical observations (i.e., location points) was compared with (i) a random set of observations having the same sample size and with (ii) an irregular distribution of observations over space. If the resulting statistic is equal to one, the events in the study area are randomly distributed. A statistic value below 1 indicates the clustered nature of the spatial distribution of observations. Statistical values above 1 highlight a uniform distribution of observations over space. Z-scores were used to ensure accuracy in the NNI testing procedure. This test specifies the difference between the mean distance from the nearest (apparent) neighbor relative to the mean distance from the nearest (random) neighbor and is calculated as follows:

$$z=\frac{{\bar{D}}_{O}-{\bar{D}}_{E}}{SE}$$

In this relation, ${\bar{D}}_{O}$ (i.e., the mean distance to the nearest neighbor point) was calculated as follows:

$${\bar{D}}_{O}=\frac{\sum_{i=1}^{n}d_{i}}{n}$$

In addition, ${\bar{D}}_{E}$ (i.e., the mean distance from the nearest neighbor assuming a random point distribution) was calculated as follows:

D¯E=0.5nA

where *n* and *A* are the number of point events and the surface area of the region, respectively; the Standard Error (*SE*) was calculated as follows:

(1) SE=0.26136n2ASE=0.26136n2A

#### 2.3.3. Natural Breaks Classification (NBC)

This algorithm is commonly used to prepare histograms and thematic maps, as well as being a basic tool for better visualization of spatial data [66,67]. NBC is considered one of the most common unsupervised methods applied to geo-visualization and spatial data analysis [68]. It provides an experimental solution for optimal delineation of homogeneous classes of a statistical distribution by minimizing the sum of the absolute standard deviation of each class. The algorithm selects an optimal set of classes calculating the absolute deviation from the class median; the observations are then transferred to the neighboring classes to reduce this error. In our study, NBC was used to produce homogeneous distance classes for the nearest neighbor collisions. Class boundaries were used as critical distances in the Knox test (see below).

#### 2.3.4. Knox Test

Being one of the most adopted tests for clustering, the Knox test allows a proper analysis of spatiotemporal interactions [68] using location (and additional attributes) of point events (road collisions in our case). The Knox test is a simple and widely used method aimed at providing a practical way to determine relevant thresholds for analysis of spatiotemporal interactions among point events. In this work, the spatiotemporal neighborhood status of the studied variable (road collisions) was evaluated on the basis of a 2 × 2 contingency table, reported below [69], outlining how two events have a spatiotemporal interaction when they are close to each other in both spatial and temporal dimensions. More specifically, this test examines the spatial and temporal neighborhood status of a given process based on a linear measure of proximity. Proximity status was determined using a neighborhood radius applied to events recorded within the same time interval. If two accidents fall within a given radius and time interval, they were considered associated over both time and space (label: 1), otherwise they were labeled as 0. In this study, distance classes were determined according to an algorithm based on optimal natural breaks (see above), discriminating classes based on the maximum intra-group similarity and the maximum inter-group differences. Natural breaks of the nearest neighbor distance in a given statistical distribution (road accidents in this case) is an appropriate technique identifying spatial thresholds of a spatiotemporal analysis according to Knox test. The Knox statistic was calculated as follows:

$$X=\overset{n}{\underset{i=1}{\sum }}\overset{n}{\underset{j=1}{\sum }}a_{ij\;}^{s}a_{ij}^{t}$$

where n is the total number of events (sample size), *δ* is a location threshold (or critical distance) and is the time threshold or critical time.

$$a_{ij}^{t}=\{\begin{array}{c} 1\;\mathrm{if}\;\mathrm{the}\;\mathrm{distance}\;\mathrm{between}\;\mathrm{cases}\;i\;\mathrm{and}\;j<??\\ 0\;\mathrm{otherwise}\;\; \end{array}$$

$$a_{ij}^{s}=\{\begin{array}{c} 1\;\mathrm{if}\;\mathrm{the}\;\mathrm{distance}\;\mathrm{between}\;\mathrm{cases}\;i\;\mathrm{and}\;j<?’\\ 0\;\mathrm{otherwise}\; \end{array}$$

$$a_{ij}^{t}=\{\begin{array}{c} 1\;\mathrm{if}\;\mathrm{the}\;\mathrm{distance}\;\mathrm{between}\;\mathrm{cases}\;i\;\mathrm{and}\;j<??\\ 0\;\mathrm{otherwise}\;\; \end{array}$$

Thresholds commonly adopted in the Knox test (see above) were used to determine at what temporal or spatial interval(s) the null hypothesis is confirmed or rejected.

## 3. Results

### 3.1. Descriptive Results

**Figure 2:**
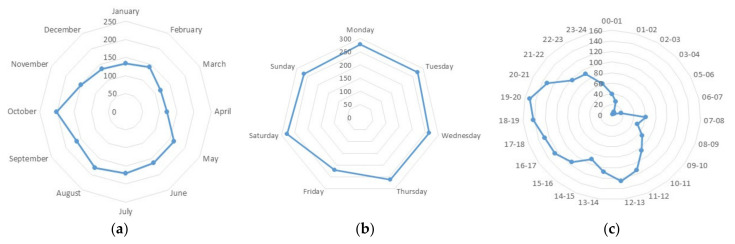
Distribution of road accidents (absolute number) in Zanjan city by month (**a**), weekday (**b**) and time (**c**).

A total of 1943 traffic accidents were recorded in Zanjan city between October 2014 and October 2015. The specific location of 100 accidents was not clear, preventing a specific investigation of these events that were removed from analysis. The spatial distribution of 1843 accidents recorded within the legal boundaries of Zanjan city was thus investigated using maps and spatial analysis. The largest number of accidents (41.6% of total collisions) occurred because of lack of attention to vehicles moving forward. Not respecting the right way was the cause of 25.7% accidents. A smaller number of collisions (6.9%) was associated with speeding violations. Sudden change of direction, reverse gear, left turn, and moving in the opposite direction were at the base of 4.9%, 4.7%, 4.0%, and 3.0% of collisions, respectively. Regarding the type of damages, 70.5% of total accidents led to (more or less) important vehicle damage. Injury-related accidents accounted for 29.3% of total accidents and 2% were fatal. The largest proportions of traffic accidents were recorded in October (10.9%), August (9.7%), and July (9.2%). As it was the beginning of school after a long summer holiday in Iran, traffic volumes increased in October, leading to a significant rise of collisions (Figure 2). The smallest proportion of traffic accidents was observed in March (6.4%).

The frequency of road accidents was also relatively stable over weekdays: the highest proportion of road accidents was recorded on both Saturday and Monday (15.1%); the lowest proportion was observed on Friday (11.9%). Closure of economic activities (factories), office centers, and businesses has intrinsically reduced the probability of having a road collision on Friday. The largest proportion of road accidents took place between 7 p.m. and 9 p.m. (8.2%). The lowest rate of collisions was observed at night, between 3 and 4 a.m. (0.1%). These results reflect the peak traffic hours in Zanjan city.

**Figure 3:**
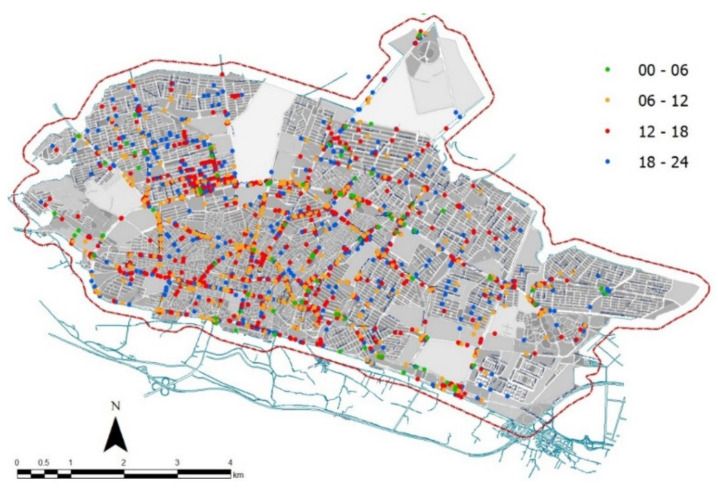
Map of road accidents in Zanjan city by time of the day including four classes that cover 6 h each, namely night (00–06), morning (06–12), afternoon (12–18) and evening (18–24).

**Figure 4:**
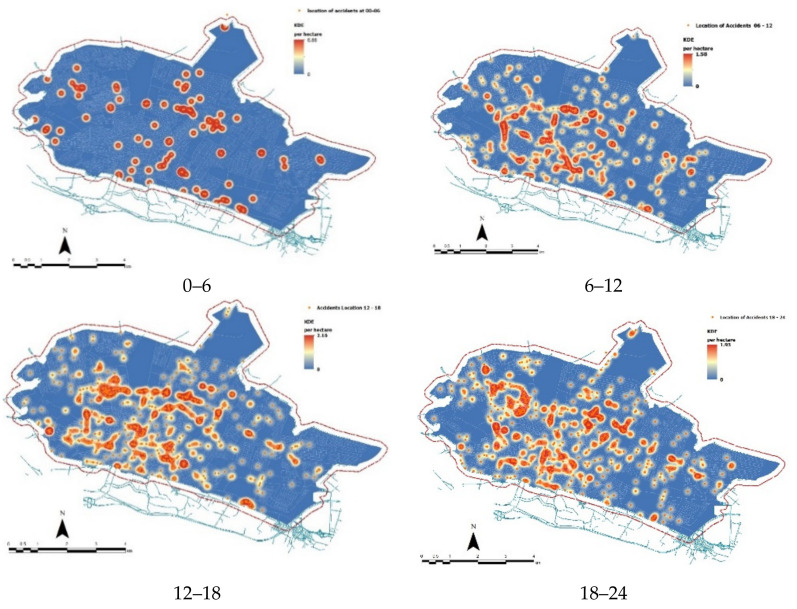
Density of road accidents (per hectare) density by time of the day (four classes covering 6 h each).

Figure 3 illustrates the spatial distribution of traffic accidents by time (four classes cumulating 6 h of the day each). A high number of accidents occurred downtown along central hours of the day (6–12 and 12–18), when businesses and office are open (Figure 4). Evening collisions (18–24) were also observed in suburban districts.

### 3.2. Spatial Analysis

Clustering (or spatial randomness) in the spatial distribution of road collisions was evaluated based on their temporal distribution along the day. Table 2 illustrates the results of a nearest neighbor analysis testing for road accidents’ clustering (or randomness) by time interval. The highest degree of spatial clustering in road accidents was observed between 12 a.m. and 6 p.m. Road accidents were clustered, displaying (i) an average distance of 79.1 m (the lowest in the sample) and (ii) a negative *z*-score (–19.8). Road accidents between 6 p.m. and 12 p.m. ranked second as far as the intensity of spatial clustering is concerned. The mean distance between accidents’ location was 85.4 m, with a z-score of –17.7. Clustering intensity decreased for morning collisions (between 6 a.m. and 12 a.m.) and night collisions (between 0 a.m. and 6 a.m.). Total accidents were definitely clustered (*z*-score = −38.1).

Descriptive statistics indicate a mean distance between the nearest neighbor car accidents of 42.8 m and a standard deviation of 50.1 m; minimum and maximum distances were 2 m and 364 m, respectively. The spatial distribution of car accidents based on the distance from the nearest neighbor collision was investigated here considering a few representative distance classes defined using the natural breaks’ algorithm. This procedure has identified 5 classes with open boundaries reported in Table 3. The largest number of collisions occurred at the smallest distance range (2–26 m). Taken together, about 75% of road collisions were recorded at a distance less than 57 m, confirming a clustered pattern of car accidents, especially at major road intersections.

**Figure 5:**
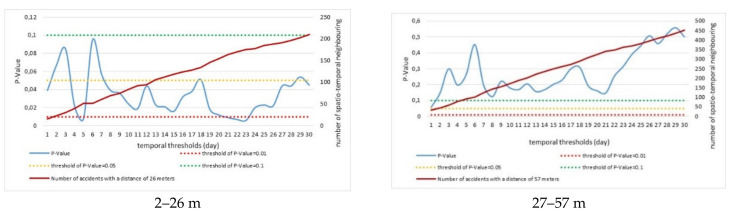

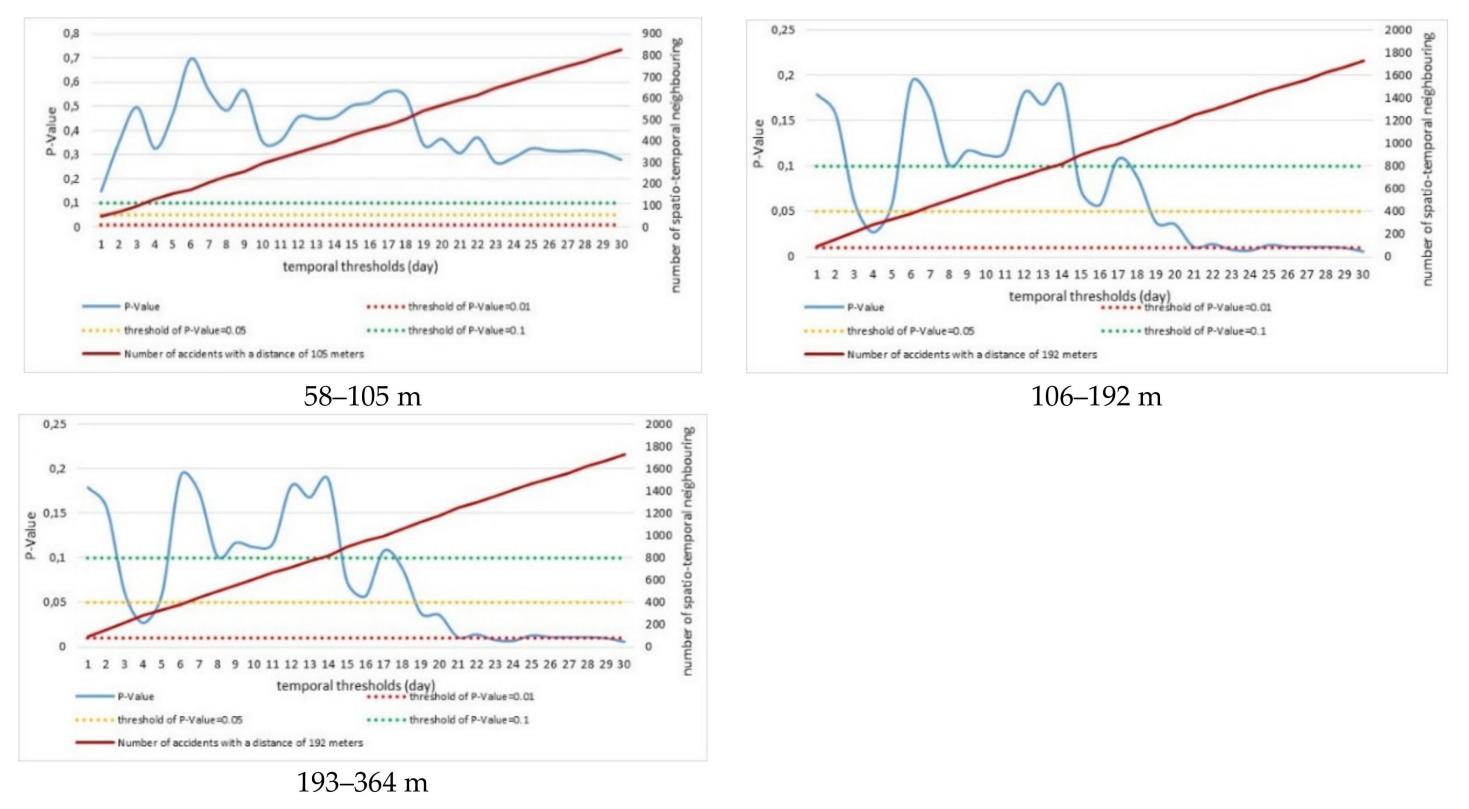
Analysis of spatiotemporal interactions in road accidents in Zanjan according to Knox test, by bandwidth range (m) and spatial threshold.

Spatiotemporal interactions of road accidents in Zanjan were illustrated in Figure 5 considering spatial thresholds (*p* < 0.1, <0.05, <0.01) that reflect three levels of significance (i.e., 90%, 95%, 99% confidence). The blue line in Figure 5 represents a continuous time trend of the probability value associated with Knox statistic against time. The values of this line below a give threshold line (green, yellow, red) indicate statistical significance of such interactions at the same confidence level. Considering a 90% confidence level, spatiotemporal interactions within a 26 m threshold were all significant for all days of the month investigated. Spatiotemporal interactions were found also significant at 95% confidence level for almost all days. A 99% confidence level was observed for spatial interactions at only four days in a month. Conversely, spatiotemporal interactions for the 57 m threshold were significant at a 90% confidence level only for one day. No significant spatiotemporal interactions were observed for the 105 m threshold. Considering the 192 m threshold, significant spatiotemporal interactions at 90%, 95% and 99% confidence levels were observed for 5, 8 and 4 days of a month, respectively. Considering the 364 m threshold, significant spatiotemporal interactions at 90%, 95% and 99% confidence level were observed respectively for 9, 9 and 0 days of a month.

Table 4 summarizes the graphical results of Figure 5 by comparing the total number of significant interactions by spatial threshold and probability level. The results indicate that the 26 m threshold had a very high difference with the remaining spatial thresholds as far as the number of significant spatiotemporal interactions is concerned. The highest frequency of road collisions car accidents was observed at this small distance range. Using a strict probability level (99% confidence level), only few interactions were significant at both small (26 m) and medium–large scale (192 m), suggesting the importance of local hotspots (e.g., large roads, traffic junctions, insertion of peri-urban motorways into the urban road network) influencing the probability of a car collision in Zanjan.

**Figure 6:**
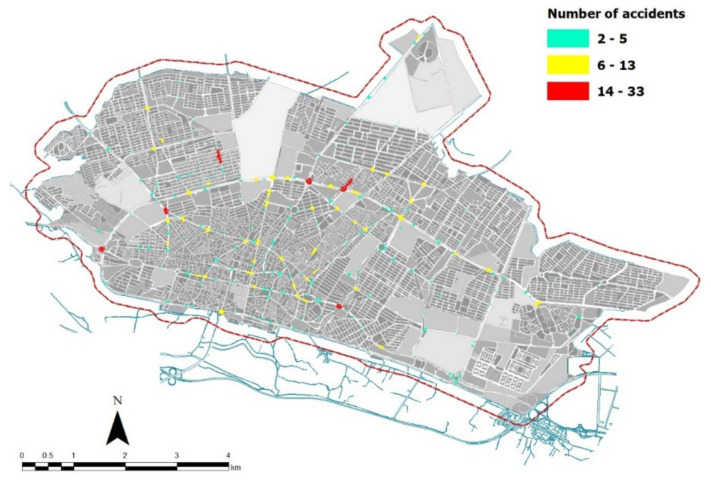
Spatial distribution of high spatiotemporal interactions of car accidents in Zanjan.

Figure 6 maps the areas with the highest number of spatiotemporal interactions in road accidents using the empirical results of Knox test. Ayatollah Dastgheib Street in Safarabad district, the Sarbaz-e Gomnam underpass on 22 Bahman axis, the intersection of Tarbiat and Southern section of Shahrak road, 17 Shahrivar intersection (i.e., Amjadieh intersection), Jihad square (West entrance of Zanjan city), and Shilat square (Moghaddam) were classified as local hotspots for road collisions, i.e., locations with the highest spatiotemporal interactions of car accidents. This means that such places have the highest frequency of road accidents at the same time of the day.

## 4. Discussion

Although recent measures of traffic regulation have been adopted in Iran at various governance levels (e.g., national, regional, local), casualties and injuries caused by vehicle accidents have increased rapidly. From this perspective, an automatic identification and classification of risky locations (i.e., local hotspots) for car accidents may contribute to informing practical actions reducing collisions and the consequent, undesired damages [70]. Geo-referenced data automatically recorded from, e.g., police, local authorities, and other public actors are a necessary information base for such methodologies. As pivotal knowledge for traffic safety management, the identification of local collision hotspots and ranking the importance of spatiotemporal interactions in car collisions can provide an accurate map of locations classified with increased risk for road accidents [71]. This information is also important for resource allocation and policy-making that contribute to mitigating health damage and economic losses derived from collisions [72]. The combined delineation of a spatial and temporal profile of road accidents is particularly appropriate information for risk classification mapping. In this regard, spatially explicit techniques seem to be relevant tools when analyzing the intrinsic characteristics of traffic crashes [73].

The present study was carried out with the aim of identifying hazardous locations of road accidents in a medium-size city in Iran, considering the space and time dimensions together. We demonstrated how a refined understanding of interactions between these two analytical dimensions may provide a complete picture of collision distribution patterns within cities, informing practical guidelines for traffic management [74]. Spatial analysis identifying local hotspots with high risk of road accidents at specific times of the day may finally contribute to more effective prevention measures [75]. To reach these objectives, a largely integrated set of spatially explicit techniques, including nearest neighbor and natural breaks algorithms and Knox test, were implemented in this study, offering a simplified procedure to analysis of spatiotemporal interactions in road accidents. This integrated strategy, and especially Knox test, seems to be more flexible than other clustering approaches because of the intrinsic ability to delineate joint spatiotemporal thresholds in the statistical distribution of collisions. The main purpose of a spatiotemporal investigation of collision interactions intrinsically assumes that a correct and complete classification of road accident hotspots requires joint identification of locations and time with the highest frequency of events [76]. 

Hotspot analysis indicates how, although properly designed, the roads with the most intense traffic load (such as the ring road, 22 Bahman road and its main intersections, Sarbaz-e Gomnam underpass, Jihad Square, and Tarbiat intersection) were locations with a particularly intense collision’s clustering. High speed, vehicle density, unregulated pedestrian ways, and car parking on the roadside were factors contributing to increased numbers of collisions. More effective traffic regulation and specific measures for reducing the risk of collisions in these locations seem to be rational measures for potentially containing the occurrence of road accidents.

The empirical results of our study also identify districts with mainly informal settlements (e.g., Islamabad (Safarabad) in the northwestern part of Zanjan) as areas with the most significant spatiotemporal interactions of traffic accidents in the study area. Such districts are particularly problematic because of traffic congestion, population density, and hyper-compact settlements. Our results clearly reconnect collision risk with external factors, among which urban design is one of the most important, and likely less studied and planned [77,78,79,80,81]. An improper design of both private and public spaces within the urban fabric, e.g., with respect to pedestrian crossings, is taken as an indirect cause of collisions in Zanjan, as well as in many other cities with the same intrinsic (morphological and functional) characteristics. Informal buildings, spontaneous settlements, unplanned infrastructures, and a persistent lack of public participation in urban management and sustainable development strategies are typical issues in cities in the Mediterranean basin and the Middle East [82,83,84,85,86]. Further investigation is necessary to better document the relationship between (unplanned) urban design and increased collision risk along road networks in (informal) metropolitan regions [87]. A comparative exercise based on a spatially explicit analysis of distribution and recurrence over time of car accidents in cities with homogeneous settlement characteristics over a representative time span [88] may definitely fill this knowledge gap. 

## 5. Conclusions

The present study demonstrated how a spatially explicit analysis may provide a complete knowledge relevant to practical strategies for traffic management, especially in cities dominated by informal settlements and deregulated urban expansion. The intrinsic development of open-access data with more precise geo-referencing of road collisions and (increasingly rich and complete) ancillary attributes will stimulate continuous improvements in statistical techniques evaluating spatiotemporal interactions in car accidents as honest (early warning) indicator of collision risk. Identification of local hotspots for road accidents, creation of maps of collision risk and a refined delineation of temporal profiles of crash events in urban areas are appropriate tools for informing strategies that may reduce (individual and community) damage caused by car accidents as a contribution to safe and sustainable urban landscapes. 

## Figures and Tables

**Table 1:** Total population of Zanjan city and urban population of the respective province, Iran in selected years between 1966 and 2016.

Population	1966	1976	1986	1996	2006	2011	2016
Zanjan city population	58,714	100,351	215,261	286,295	349,713	386,851	430,871
Annual population growth rate (%)	–	5.5	7.9	2.9	2	2	2.2
Urban population (Zanjan province)	82,599	144,612	317,113	429,013	559,340	634,809	711,177
Zanjan city in total urban population	71.1	69.4	67.9	66.7	62.5	60.9	60.6

Source: Statistical Centre of Iran, 1966–2016.

**Table 2:** Results of a nearest neighbor test by time interval.

Interval	Number	Mean Distance (m)	Expected Distance (m)	Nearest Neighbor Rate	*Z*- Score	*p*- Value	Clustering Rank
Accidents 0 a.m.–6 a.m.	91	241.6	359.5	0.67	−5.98	<0.001	4
Accidents 6 a.m.–12 a.m.	394	111.0	172.8	0.64	−13.57	<0.001	3
Accidents 12 a.m.–6 p.m.	665	79.1	130.3	0.61	−19.79	<0.001	1
Accidents 6 p.m.–12 p.m.	693	85.4	133.0	0.64	−17.66	<0.001	2
Total accidents	1843	42.8	79.9	0.54	−38.13	<0.001	–

**Table 3:** Distribution of road accidents by distance to the nearest traffic accident (distance classes were determined according to the natural breaks criterion.

Class of Nearest Distance (m)	Number of Collisions	Per Cent Share in Total Accidents
2–26	1017	55.18
27–57	375	20.35
58–105	255	13.84
106–192	152	8.24
193–364	44	2.39

**Table 4:** Per-month significant accidents’ interactions by spatial threshold and probability level.

Upper Bandwidth Threshold (m)	Number of Significant Interactions by Confidence Level (out of 30 Total Interactions)		
	*p* < 0.1	*p* < 0.05	*p* < 0.01
26	30	24	4
57	1	0	0
105	0	0	0
192	5	9	4
364	9	9	0

## Data Availability

Some elaborations presented in this study are available on reasonable request from the corresponding author; raw data cannot be disseminated since they are not publicly available for security reasons.
